# Supplementary material for: Evaluation of Qualitative Dietary Protocol (Diet4Hashi) Application in Dietary Counseling in Hashimoto Thyroiditis: Study Protocol of a Randomized Controlled Trial
Source: Int J Environ Res Public Health. 2019 Dec 2;16(23):4841. doi: 10.3390/ijerph16234841 (PMC6926951; doi:10.3390/ijerph16234841)
Supplement: Supplementary file 1 [file ijerph-16-04841-s001.pdf]

International Journal of
Environmental Research and Public Health

MDPI

Supplementary material

Table S1. Leaflet with general dietary guidelines (in English).

General dietary guidelines

There are guidelines that you should follow every day.

1. **Eat meals regularly**

Eat a few small meals every day, at regular times. Always start the day with breakfast.

2. **Take care of hydration**

Every day, drink an adequate amount of non-sweetened drinks, preferably water, up to 2 litres a day. Drink regularly throughout the day, in small amounts. Try to limit the intake of strong tea and coffee.

3. **Don't overeat**

Eat as much as you need, but do not consume excessive amounts of foods in one meal.

4. **Cook 'delicately'**

Try to eat delicately cooked foods – boil in water or steaming, bake in a sleeve, use stewing at a low temperature, with a small amount of fat. Avoid frying, baking or stewing at too high a temperature. Add good-quality fats to your dishes, preferably raw. Take care of the consumption of unrefined oils, e.g. cold-pressed.

5. **Don't over-salt**

Try to avoid excessive salt. Limit the consumption of hidden salt sources (e.g. canned food, pickles), ready-to-eat foods, seasonings with monosodium glutamate, ready-to-eat sauces, broth cubes, etc. Replace the salt with natural herbs, fresh or dried, and spices.

6. **Read labels**

Pay attention to the labels on the foods you buy. Avoid sweetened, salted foods and hardened vegetable fats, e.g. margarine in a cube that is mainly found in processed foods, e.g. biscuits. Pay attention to the food composition. The ingredient that is listed first is in the largest quantity.

7. **Take care of physical activity**

The daily amount of moderate and regular physical activity is very important. This includes a brisk walk, cycling or daily activities in the house.

Table S2. Leaflet with general dietary guidelines (in Polish).

Ogólne zalecenia dietetyczne

Poniżej znajdują się zalecenia, które należy wprowadzić na co dzień

1. **Jedz regularnie posiłki**

Codziennie jedz regularnie kilka niedużych posiłków, o stałych porach. Zawsze zaczynaj dzień od śniadania.

2. **Dbaj o nawodnienie**

Codziennie pij odpowiednią ilość niesłodzonych napojów, najlepiej wody, nawet 2 litry na dobę. Pij napoje regularnie w ciągu całego dnia, małymi porcjami. Staraj się ograniczyć spożycie mocnej herbaty i kawy.

3. **Nie przejadaj się**

Jedz tyle ile potrzebujesz, ale nie przesadzaj z nadmierną ilością żywności w jednym posiłku.

4. Gotuj 'delikatnie'
Staraj się jeść potrawy poddane delikatnej obróbce termicznej – gotuj w wodzie lub na parze, piecz w rękawie, stosuj duszenie w niskiej temperaturze, z małą ilością tłuszczu. Unikaj smażenia, pieczenia lub duszenia w zbyt wysokiej temperaturze. Dodawaj do potraw tłuszcze dobrej jakości, najlepiej na surowo. Zadbaj o spożycie olejów nierafinowanych, np. tłoczonych na zimno.

5. Nie przesalaj
Staraj się unikać nadmiernej ilości soli. Ogranicz spożycie ukrytych źródeł soli (np. konserw, pikli), żywności gotowej do spożycia, przypraw z glutaminianem sodu, gotowych sosów, kostek rosołowych, itp. Zastąp sól naturalnymi ziołami, świeżymi lub suszonymi oraz przyprawami korzennymi.

6. Czytaj etykiety
Zwracaj uwagę na etykiety żywności, którą kupujesz. Unikaj żywności słodzonej, solonej i utwardzonych tłuszczów roślinnych, np. margaryn w kostce, które znajdują się głównie w przetworzonej żywności, np. herbatnikach. Zwracaj uwagę na skład żywności. Składnik, który jest wymieniony jako pierwszy, znajduje się w największej ilości.

7. Zadbaj o ruch
Codzienna porcja umiarkowanej i regularnej aktywności fizycznej jest bardzo ważna. Wystarczy intensywny spacer, jazda rowerem lub codzienna aktywność w gospodarstwie domowym.

Table S3. The qualitative dietary protocol: (A) Self-monitoring diary for recommended foods (in Polish).

**Żywność rekomendowana do spożycia**<br>Kontroluj częstotliwość spożycia żywności po każdym posiłku/epizodzie żywieniowym (lub co najmniej raz dziennie) i sprawdzaj zgodność z rekomendacjami.<br>Wskazano **minimalną** częstotliwość spożycia.							
Dzień	**Warzywa** (patrz dziennik B)	**Żywność bogata w wapń** np. mleko, jogurt, kefir, twaróg, ser	**Owoce**	**Pełnoziarniste produkty zbożowe** np. kasza gryczana, razowe pieczywo pszenne i żytnie	**Żywność pochodzenia zwierzęcego bogata w cynk** np. mięso: wołowe, jagnięce, wieprzowe; jaja	**Żywność pochodzenia zwierzęcego bogata w selen** np. ryby, owoce morza	**Orzechy i ziarna** np. orzechy różne, ziarna dyni, ziarna słonecznika
	Kilka razy dziennie	Kilka razy dziennie	Raz dziennie	Raz dziennie	Kilka razy w tygodniu	Kilka razy w tygodniu	Kilka razy w tygodniu
Miesiąc:	**Zaznacz × za każdym razem po spożyciu żywności**						
1							
2							
3							
…							
31							
Miesiąc: Czerwiec 2019	***Przykładowy zapis Pacjenta***						
1	××	×	××	×		×	
2	×××	×	×	×	×	×	
3	×	×××	×	×		×	
4	×	××		×	×	×	
5	×××	×	×	×	×	×	
6	××	××				×	
7	×××	××	×		×		

Table S4. The qualitative dietary protocol: (B) Self-monitoring diary for foods with limited consumption (in Polish).

**Żywność, której spożycie należy ograniczać**<br>Kontroluj częstotliwość spożycia żywności po każdym posiłku/epizodzie żywieniowym (lub co najmniej raz dziennie) i sprawdzaj zgodność z rekomendacjami.<br>Wskazano **maksymalną** częstotliwość spożycia.						
Dzień	**Warzywa krzyżowe na surowo**<br>np. kapusta: włoska, pekińska, biała, czerwona, brokuły, brukselka, kalafior, jarmuż	**Słodycze, cukier i miód,**<br>w tym wysoko słodzone dżemy, kandyzowane owoce, itp.	**Napoje słodzone i energetyzujące**<br>np. z cukrem, syropem glukozowo-fruktozowym, słodzikami	**Żywność typu fast food**<br>np. hot-dogi, hamburgery, czipsy, frytki, pizza, żywność smażona w głębokim tłuszczu	**Soja i proso**<br>np. sojowe: nasiona, kiełki, tofu, mleczko; kasza jaglana	**Alkohole**<br>np. wino, piwo, drinki, wódka, brandy
	Raz w tygodniu	Raz w tygodniu	Raz w tygodniu	Raz w tygodniu	Dwa razy w miesiącu	Raz w miesiącu
Miesiąc:	**Zaznacz × za każdym razem po spożyciu żywności**					
1						
2						
3						
…						
31						
Miesiąc: Czerwiec 2019	***Przykładowy zapis Pacjenta***					
1	×					
2			×			
3	×					
4						
5		×				
6						
7			×	×	×	
